# Expression and Activation of STAT3 in the Astrocytes of Optic Nerve in a Rat Model of Transient Intraocular Hypertension

**DOI:** 10.1371/journal.pone.0055683

**Published:** 2013-01-31

**Authors:** Shaodan Zhang, Weiyi Li, Wenqian Wang, Samuel S. Zhang, Ping Huang, Chun Zhang

**Affiliations:** 1 Department of Ophthalmology, Peking University Third Hospital, Peking University Eye Center, Beijing, China; 2 Department of Ophthalmology, the 4th People's Hospital of Shenyang, Shenyang Institute of Ophthalmology, Liaoning, China; 3 Department of Neural and Behavioral Sciences, Penn State University, Hershey, Pennsylvania, United States of America; 4 Singapore Eye Research Institute, Singapore National Eye Centre, Singapore, Singapore; Massachusetts Eye & Ear Infirmary, Harvard Medical School, United States of America

## Abstract

Lamina cribosa, an astrocyte-rich region, is the origin of axonal degeneration in glaucomatous neuropathy. Astrocytes are particularly activated during optic nerve (ON) degeneration and are likely to contribute to the pathogenesis of glaucomatous optic neuropathy. Signalling mechanisms that regulate different aspects of astrocyte reactiviation in response to intraocular hypertensive injury are not well defined. Signal transducer and activator of transcription protein-3 (STAT3) is a transcription factor that participates in many biological processes and has been implicated as activator of reactive astrogliosis. In this study, we investigated the role of STAT3 in regulating the activation of astrocytes to transient intraocular hypertension *in vivo* by using a rat ocular hypertension model. ON astrocytes hypertrophy was observed early after intraocular hypertensive stress. Morphological changes in glial fibrillary acidic protein (GFAP) positive cells coupled with axon loss in the optic nerve was detected at day 7 after the injury. Nestin was significantly upregulated in ON astrocytes as early as day 2 post injury and kept elevated through post injury day 7. Phosphorylated STAT3 (*p*STAT3) was markedly upregulated in ON astrocytes at post injury day 1, prior to the reactivation of ON astrocytes. These findings indicate that STAT3 signalling is involved in the initiation of astrocyte reactivation in optic nerve injury.

## Introduction

Glaucomatous optic neuropathy is characterized by the pattern degeneration of retinal ganglion cells (RGCs) and their axons, which results in irreversible visual field loss [1]. Elevated intraocular pressure (IOP) is the most recognized glaucoma risk factor. IOP reduction is one of the major managements of glaucoma therapy. However, glaucoma neuropathy is still progressing in normal tension glaucoma patients and some hypertensive patients with well controlled IOP. The molecular mechanisms of glaucomatous optic nerve degeneration and RGC loss are not fully understood.

Lamina cribosa, an astrocyte-rich region, is known to be the origin of axonal degeneration in glaucomatous neuropathy [2], [3], [4], [5], [6], [7]. Astrocytes are the predominant cell type in the optic nerve head. In addition to provide homeostatic support to axons, the ON astrocytes also play an important role in the mechanical stability of the lamina cribrosa [8], [9].

Most studies had focused on astrocytes located within the lamina cribosa region of the optic nerve head. Many reports have provided evidence that astrocytes are responsible for many pathological changes in the glaucomatous optic nerve head (ONH) [10]. Astrocytes reactivation is observed during optic nerve degeneration and is likely to contribute to the pathogenesis of glaucomatous optic neuropathy [11]. Recently, Marsh-Armstrong described the changes of ON glial populations in ON degeneration model in DBA/2J mice [2]. They found a marked activation in astrocyte occurred early in the degeneration process. The loss of oligodendrocytes and activation of microglia occurred late after the significant loss of axons and RGC bodies. Thus, modulating the astrocytes activation is crucial to control astrogliosis, therefore be a potential therapeutic strategy for the treatment of glaucomatous neuropathy. The progressive activation of ON astrocytes is thought to be finely regulated by the complex intercellular and intracellular signalling mechanisms [12]. Understanding the detail of the signalling mechanisms would benefit of developing new therapeutic strategies for glaucomatous optic neuropathy.

Signalling mechanisms that regulate astrocyte activation in glaucomatous optic neuropathy are not well defined. The signal transducer and activator of transcription 3 (STAT3) is a member of the Jak-STAT signalling family. It transduces signals for a group of cytokines and growth factors implicated in the acute response. Our previous study described the expression and activation profile of STAT proteins during mouse retina development [13]. There is evidence that JAK-STAT3 signalling regulates proliferation of astrocytes *in vitro* [14], [15]. The activation of STAT3 by phosphorylation increased markedly in astrocytes, microglia, endothelial cells, and neurons shortly after CNS stress [16], [17], [18]. Some studies have reported STAT3 signalling pathway is critical transducers of certain aspect of reactive astrogliosis in spine injury [19], [20].

In order to determine the role of STAT3 signalling in the reactivation process of ON astrocytes *in vivo*, we investigated the relationship between the activation of STAT3 signalling and the response of optic nerve astrocytes in the ischemia-reperfusion injury model created by transient intraocular hypertension.

## Materials and Methods

### Animals

Forty-five female Wistar rats (8 week- old, 200–250 g), were housed in temperature-controlled rooms with a 12 h light/dark cycle and free access to food and water. All animal handling protocols are in accordance with ARVO Statement for the Use of Animals in Ophthalmic and Vision Research and were approved by the Institutional Animal Care and Use Committee of Peking University Health Science Center.

We adopted the previously reported transient intraocular hypertension model [21], [22] with minor modification. Briefly, animals were anesthetized with intraperitoneal injection of ketamine (10 mg/kg) and xylazine hydrochloride (5 mg/kg). Topical tropicamide-phenylephrine ophthalmic solution (Santen Pharmaceutical Cor., Ltd. Japan) was applied to achieve mydriasis. The anterior chamber of the right eye was then cannulated with a 30-gauge needle connected to a sterile saline-filled bottle. IOP was maintained at 110 mmHg for 60 minutes by lifting the bottle to a height of 1496 mm above the eye. Retinal ischemia was confirmed by whitening of the fundus. Only animals with unimpaired lenses and corneas after surgery were enrolled in further study.

At day 1, 2, 3 and 7after the transient intraocular hypertensive stress, rats were euthanized. Retinas and optic nerves were harvested immediately for immunohistochemistry and western blot observation. Retina and optic nerves from the untreatment animals were used as control.

### HE staining and immunohistochemistry

Three rats in each group were transcardially perfused with 250 mL 0.01 M phosphate saline buffer (PBS, pH 7.4), followed by 100 ml ice-cold 4% (w/v) paraformaldehyde. Eyes were enucleated with 1–2 mm optic nerve attached. Samples were post-fixed in 4% paraformaldehyde for 8 hours, followed by cryoprotection in 30% sucrose. Optic nerve was dissected from the globe before embedding. Cryostat sections of the retina (10 µm, sagittal) and optic nerve (8 µm, transversal) were mounted onto poly-L-lysine coated slides. HE staining was performed by the standard protocol. For immunofluorence staining, optic nerve sections were blocked in 5% bovine serum albumin in PBST for 1 hour at room temperature and then incubated with mouse anti-GFAP (1∶1000; DAKO, Glostrup, Denmark)/rabbit anti-STAT3(1∶100; Santa Cruz Biotechnology, Santa Cruz, CA), rabbit anti-GFAP(1∶1000; DAKO, Glostrup, Denmark)/mouse anti-βIII tubulin (1∶1000; Abcam, Cambridge, MA, USA), rabbit anti-STAT3 (1∶1000; Santa Cruze)/mouse anti-βIII tubulin (1∶1000; Abcam), mouse anti-GFAP (1∶250; EMD Millipore, Billerica, MA)/rabbit anti-pSTAT3(1∶250; Santa Cruz Biotechnology,Santa Cruz, CA), or rabbit anti-GFAP(1∶1000; DAKO)/mouse anti-nestin(1∶1000; Chemicon, Temecula, CA) respectively, overnight at 4°C. After 5 min/wash in PBST for 3 times, the sections were incubated with the appropriate secondary antibodies that consisted of Alexa Fluor 488 goat anti-rabbit IgG (H+L) (1∶1000; Invitrogen, Eugene, OR, USA) and Alexa Fluor 594 goat anti-mouse IgG (H+L) (1∶1000; Invitrogen). DAPI was applied to demonstrate to cell nucleus. Fluorescence signals were visualized by confocal fluorescence microscopy (Carl Zeiss Inc., Germany).

For phosphorylated STAT3 (p-STAT3) staining, the sections were treated with 0.3% H_2_O_2_ for 10 min to quench endogenous peroxidase activity. Nonspecific binding sites were blocked with 5% bovine serum albumin in TBST for 1 hour. Then, sections were incubated with rabbit polyclonal anti-p-STAT3 (1∶100; Cell Signalling Technology, Danvers, USA) in TBST overnight at 4°C. After extensive wash in 0.01 M TBST, sections were incubated with biotinylated anti-rabbit IgG for 2 hours at RT followed by avidin-biotin-peroxidase complex incubation for another 2 hours. Positive signals were visualized using DAB (diaminobenzidine tetrahydrochloride) method. Images were captured by Leica CCD color camera.

### Western blot analysis

At day 1, 2, 3 and 7 after the transient intracoluar hypertensive stress, treated and control animals (n = 6 in each group) were euthanized by an overdose of urethane. 2 mm post-laminar optic nerves were collected and frozen in liquid nitrogen immediately. Each western blot sample contained three optic nerves.

Tissues were lysed in ice-cold RIPA buffer containing protease and phosphatase inhibitor. Lysates were centrifuged for 15 min at 12000 g at 4°C, and supernatants were assayed for protein concentration. Equal amount of 50 µg total proteins was separated by SDS-polyacrylamide gel electrophoresis and transferred onto PVDF membranes. The membrane was blocked with 5% non-fat dry milk in TBST and subsequently incubated with p-STAT3(rabbit polyclonal, 1∶200, Cell Signalling Technology), STAT3 (rabbit polyclonal, 1∶200, Santa Cruze) and GFAP(rabbit polyclonal, 1∶2000, DAKO) overnight at 4°C. GAPDH (mouse monoclonal, 1∶1000, Chemicon, Temecula, CA) was used as an internal control. The proteins were detected by IRDye 800CW goat anti-rabbit or goat anti-mouse secondary antibodies (Rockland Immunochemicals) and visualized with Odyssey Infrared Imaging System (LI-COR Biosciences).

### Data analysis

Images of three sections from each optic nerve were captured and were exported into ImageJ (National Institutes of Health) software for further analysis. β- tubulin or GFAP-positive areas measured and the percentage these positive areas to the total cross-sectional surface area of the optic nerve were calculated. In STAT3-labeled sections, the width of primary astrocyte process was measured at 12–15 µm from the margin of the nucleus for at least 10 cells. P-STAT3 positive cells' counting was manually performed at 200× magnification within the counting frame. For western blot analysis, protein expression level of GFAP, STAT3 and p-STAT3 was determined by its optical density value normalized by GAPDH.

Statistical analyses were performed using SPSS 11.0 software. For all measurements, results from three rats in each group were expressed as mean±SD. Differences between experimental and control groups were tested using an ANOVA test. p<0.05 is considered as statistically significant.

## Results

### 1. Different responses of GFAP expression in the retina and optic nerve after transient intraocular hypertension

**Figure 1:**
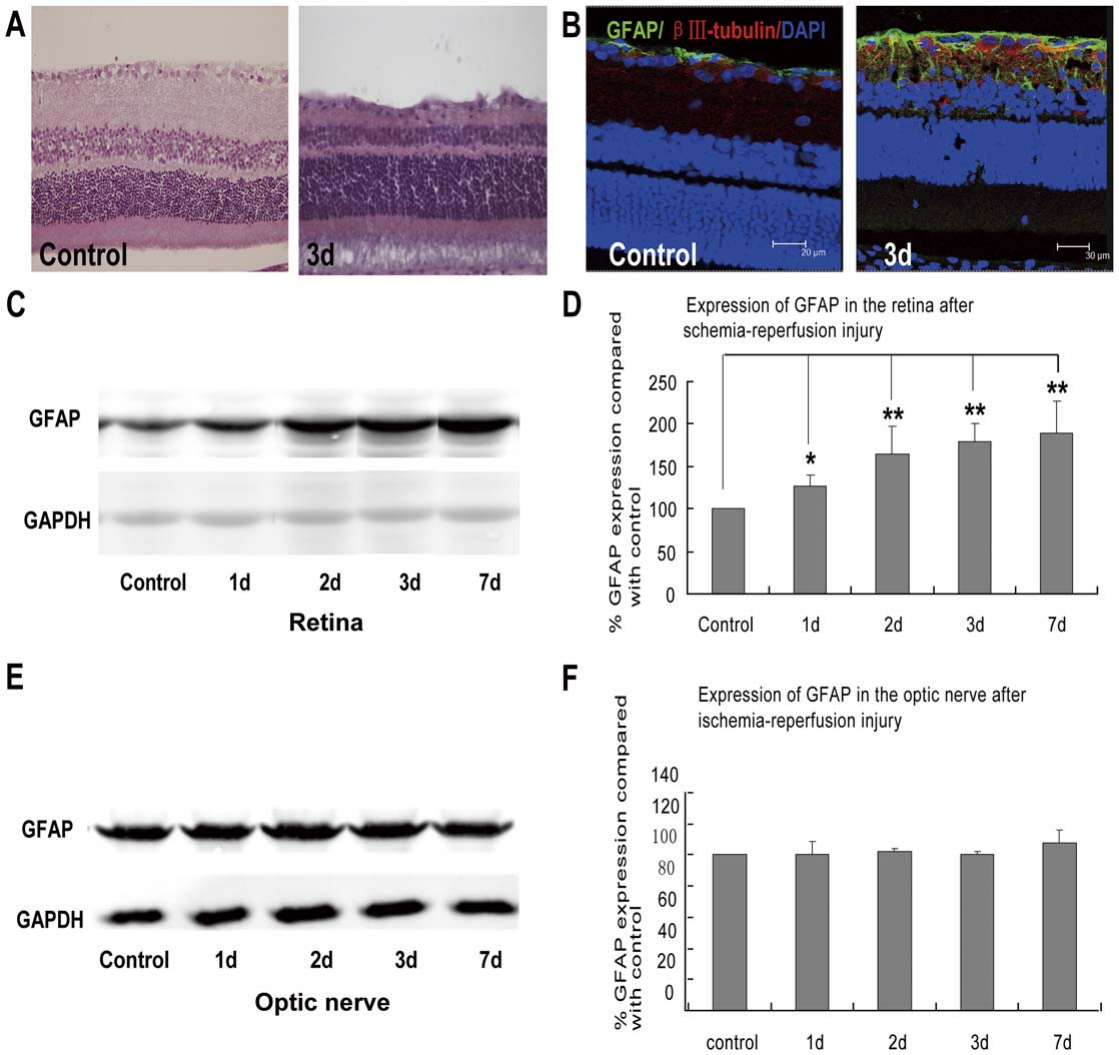
Different GFAP expression between retina and optic nerve after the induction of transient intraocular hypertension. (**A**) HE staining showed that comparing with the intact and regular retina in control rats, transient intraocular hypertension induced significant decrease in the thickness of retina as early as 3 days. (B) In control retina, GFAP immunoreactivity was confined to the nerve fiber layer (NFL) and ganglion cell layers. Three days after the induction of transient intraocular hypertension, GFAP-labeled processes were found to enwrap the RGCs and extend throughout the entire retina. (C–D) Immunoblots revealed increased expression of GFAP in the retina at 1, 2, 3, and 7 days after the injury. (E–F) While in the optic nerve, the expression of GFAP seemed to be constant throughout the observation course. * p<0.05; ** p<0.01.

In the transient intraocular hypertension group, we observed a significant decrease in retina thickness, more prominent in the inner plexiform layer especially at day 3. Morphological changes of astrocytes were also noticed. In control retinas, the limited GFAP immunofluorescence was confined exclusively to the nerve fiber layer (NFL) and the retinal ganglion cell layer (RGCL). Transient intraocular hypertension induced GFAP elevation was observed in the entire retina and was persistent throughout the tested window. Consistently, western blot analysis revealed 1.26, 1.63, 1.78 and 1.89 folds increase in the retinal GFAP expression at post-injury day 1,2,3 and 7, respectively, compared with controls. However, GFAP expression in optic nerve had no difference between the treated and control groups in all the tested time points. These findings suggested that glial response to transient intraocular hypertension injury may be different between retina and optic nerve. (Fig. 1)

### 2. Axonal loss and morphological changes of astrocytes in the optic nerve after transient intraocular hypertension

**Figure 2:**
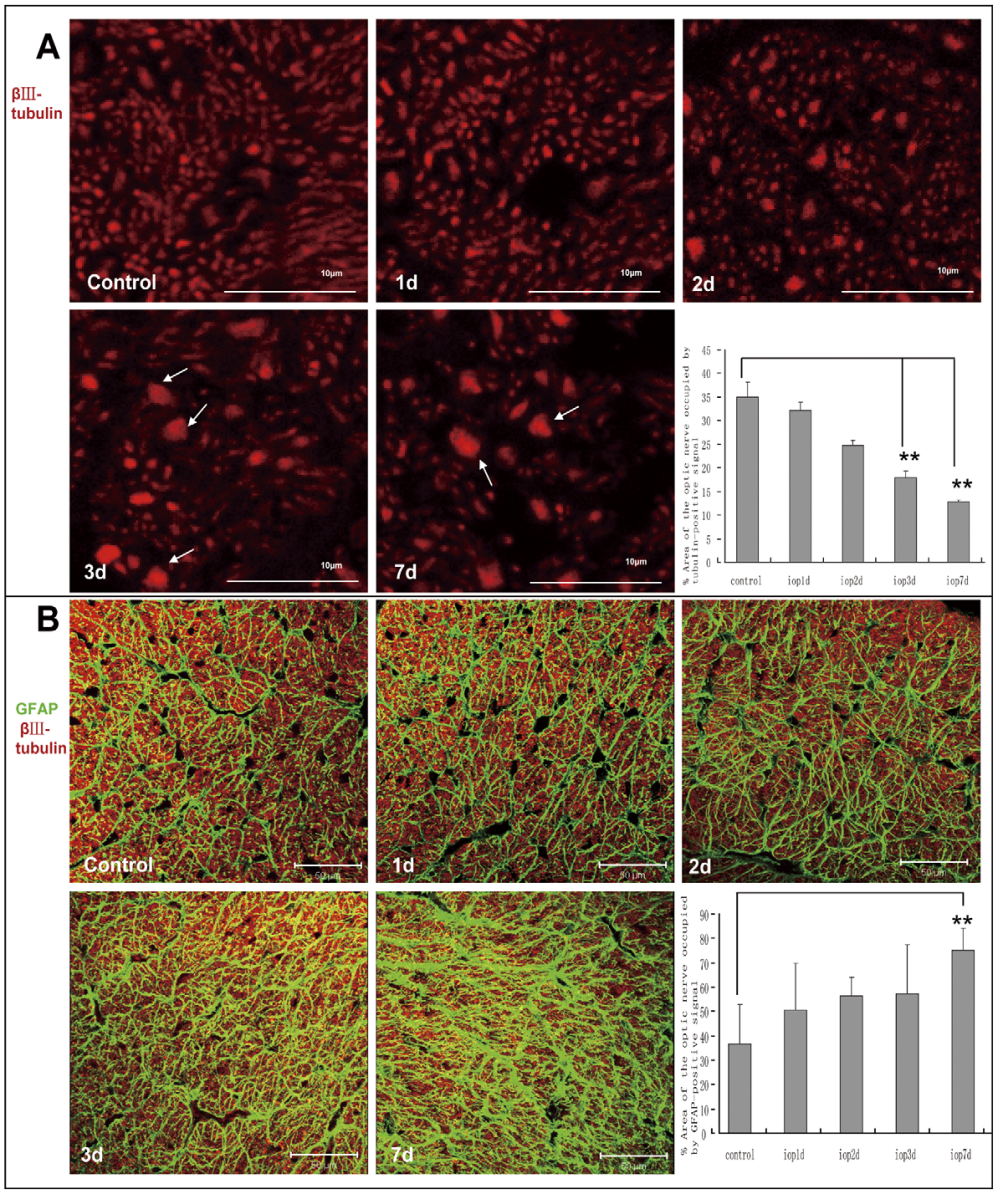
Transient intraocular hypertension induced progressive RGC axon loss and structural remodeling of the astrocytes in the optic nerve. (A) Transverse section of control optic nerve was consisted of bundles of tiny and homogeneous RGC axons. From 2 days after the induction of transient intraocular hypertension, progressive axon loss accompanied with fused axonal mass was observed. Quantitative analysis revealed significant decrease in the βIII-tubulin positive area in the cross section of optic nerve at 3 and 7 days. Scar bars, 10 µm. (B) Within normal optic nerve, thin and long primary processes of astrocytes transversely connected with each other to form a honeycomb appearance. These glial tubes segregated neighboring axons into bundles. GFAP and βIII-tubulin double negative spaces were found scattering among the axon bundles, representing the glial cell bodies and vessels. Two days after injury, processes of the astrocytes became much thicker and denser. Spaces among bundles still could be clearly seen. While at 7 days, reactive astrocytes showed hypertrophy and disorganization of their processes. Spaces among bundles were nearly undetectable. Morphological analysis showed that GFAP-positive area in the optic nerve increased gradually following the induction of transient intraocular hypertension and reached statistical significance at 7 days. Scar bars, 50 µm.

To investigate whether retina and optic nerve possessed different neuronal and glial responses to the transient intraocular hypertension injury, transversal sections of optic nerve were immunostained with βIII-tubulin and GFAP to illustrate the changes of RGC axon and astrocytes respectively. Normal ON structure was maintained until post-injury day 2. The scatter GFPA/βIII-tubulin positive spots were then detected among normal axons, which suggested the fusion and dying of the axons. At day 3 and 7, significant loss of RGC axons was also noticed. The Number and diameter of the axon mass increased markedly. Quantitative analysis showed area of βIII-tubulin positive signal progressively decreased in the optic nerve of (24.8±1.0)%, (17.9±1.3)% and (12.9±1.0)% at day 2, 3 and 7 respectively, comparing (35.1±3.0)% in controls. (Fig. 2A) Astrocytes hypertrophy was observed in the same region along with the neuronal damage. In the cross section of normal optic nerve, astrocytes with thin and long processes connected together forming a dense glial honeycomb. These processes divided adjacent RGC axons into bundles. GFAP-negative spaces representing the cell bodies of astrocytes and some large blood vessels were scattering in between these axon bundles. Initially, GFAP-positive processes made up to (36.6±16.3)% of the total area of the optic nerve section. The glial change was advanced through the tested window. At day 7, thick and disorganized astrocytes occupied (75.1±9.2) % of the total area of the optic nerve section, making the spaces among bundles hardly detectable. (Fig. 2B)

### 3. Expression of STAT3 in the astrocytes of normal and transient intraocular hypertension-injured optic nerve

**Figure 3:**
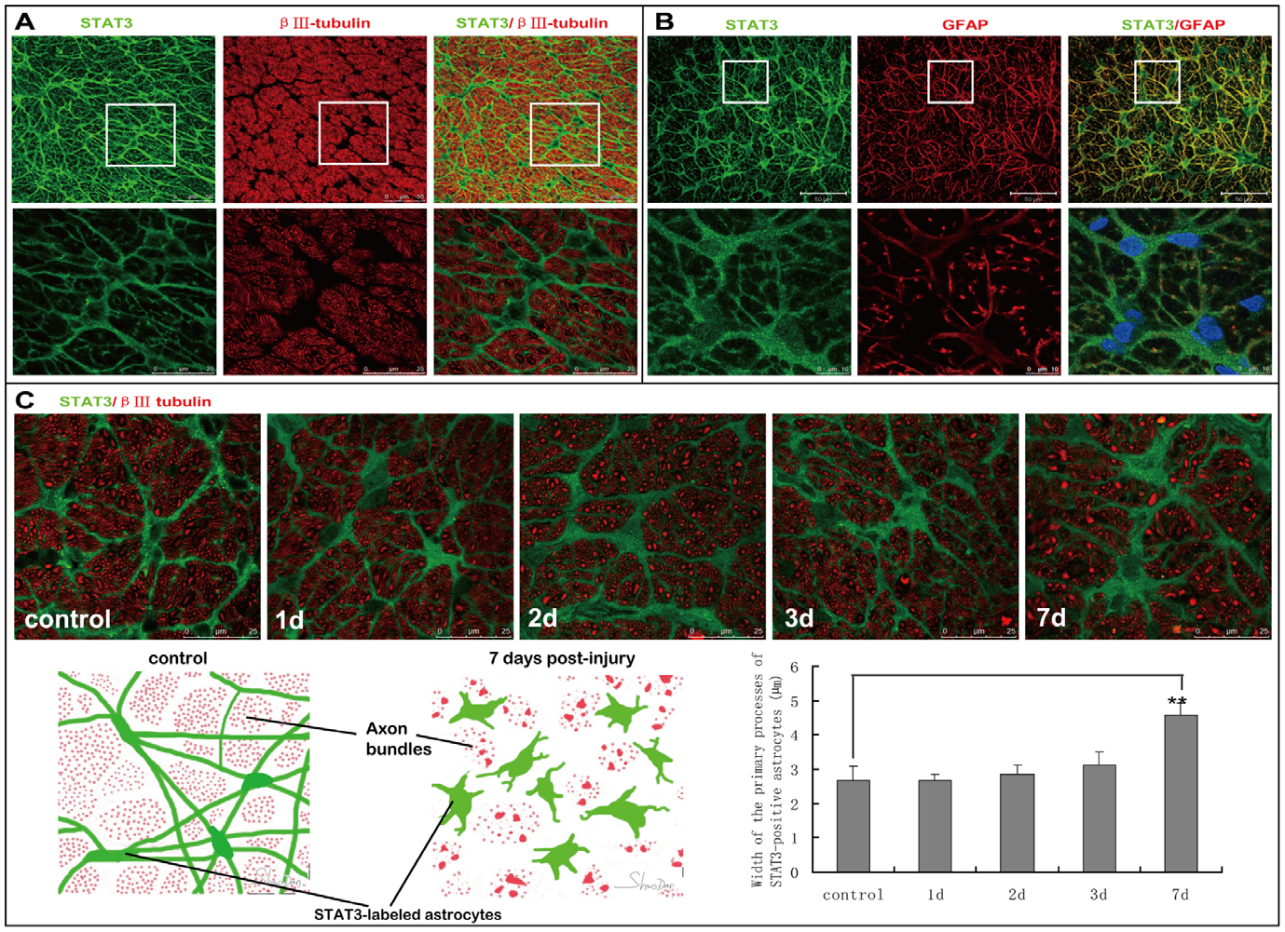
Expression of STAT3 in the astrocytes of normal and transient intraocular hypertension-injured optic nerve. (A) In control rats, STAT3 immunoreactivity was observed in the processes and perinuclear cytoplasm of astrocyes. STAT3-positive processes connected to form an appearance of honeycomb and partitioned neighboring RGC axons into bundles. Spaces among these axon bundles were perfectly filled by STAT-3 positive cell body. (B) STAT3 showed good co-localization with GFAP in the processes of astrocytes. However, STAT3-positive signal could also be observed at the perinulear cytoplasm, where no GFAP immunoreactivity was found. Therefore STAT3 may give clearer and more entire appearance of the astrocytes than GFAP. (C) From day 3 after the induction of transient intraocular hypertension, STAT3-labeled primary processes of astrocytes in the optic nerve became thicker and tortuous,destroying normal regular honeycomb architecture of the glias. At day 7, hypertrophy of cell soma, thickening of primary processes, and retraction of higher-order processes in STAT3-positive astrocytes were easily identified. The schematic in **C** depicted how the morphology of STAT3-positive astrocytes changed following transient intraocular hypertension. Quantitative analysis showed that average thickness of STAT3-labeled primary processes was 4.58±0.36 µm at day 7 post-injury compared with 2.68±0.41 µm in normal optic nerve.

In control rats, STAT3-labeled cells with long and thin processes were transversely oriented in the cross section of optic nerve. These processes overlapped extensively, giving architecture of honeycomb and dividing RGC axons into bundles. STAT3-positive signals could also be observed in perinuclear cytoplasm in addition to processes. STAT3-labeled cell body filled the spaces among axon bundles. Transient intraocular hypertension led to obvious morphological changes of STAT3-positive astrocytes in the optic nerve. Comparing with the delicately organized glial connection in control optic nerve (mean diameter of primary processes, 2.68±0.41 µm), STAT3-labeled primary processes of astrocytes became thicker and tortuous ever since post-injury day 3 (mean diameter of primary processes, 3.12±0.37 µm), which progressively interrupted the glial honeycomb. At day 7, hypertrophy of cell body, thickening of primary processes, and retraction of higher-order processes in STAT3-positive astrocytes became more prominent (mean diameter of primary processes, 4.58±0.36 µm). Schematic representation of STAT3-positive astrocytes changes following transient intraocular hypertension was shown in Fig. 3C.

### 4. Expression of p-STAT3, the activated form of STAT3, in the reactive astrocytes in optic nerve after transient intraocular hypertension

**Figure 4:**
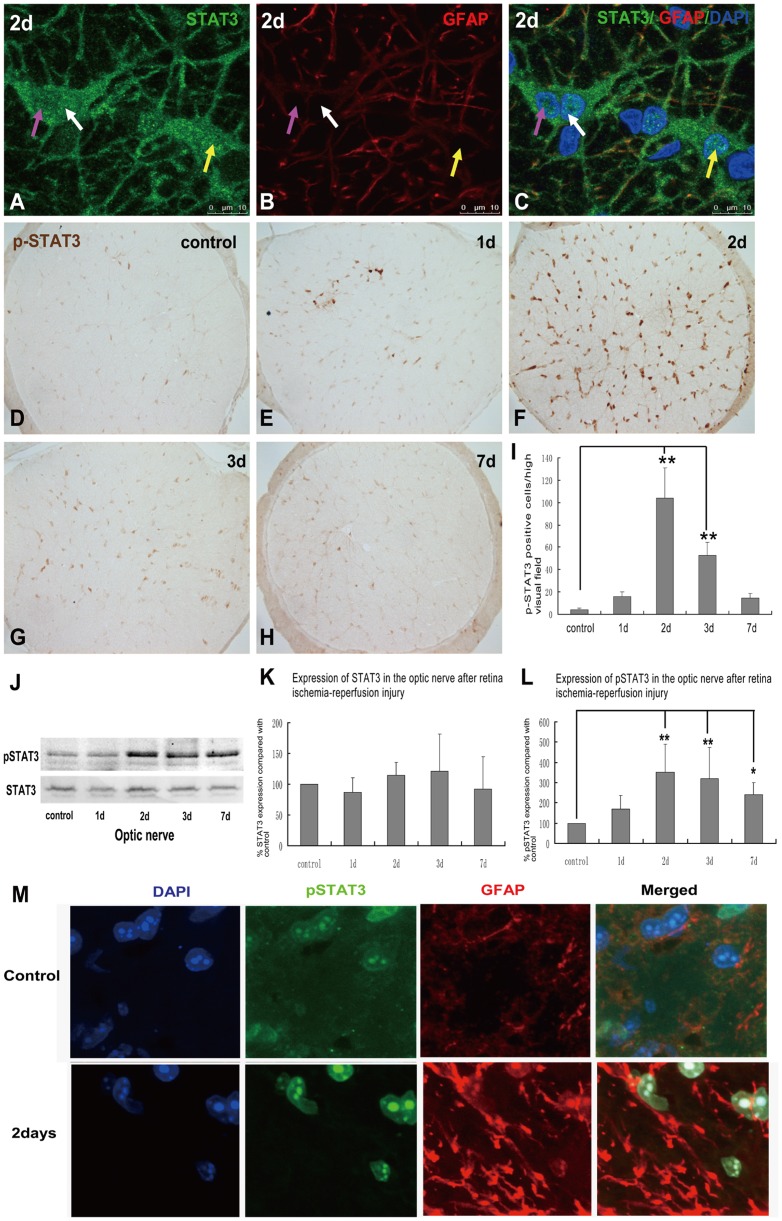
Expression of pSTAT3 in control and transient intraocular hypertension injured optic nerve. Nuclear translocation of STAT3 was observed in the optic nerve at 2 days after the induction of transient intraocular hypertension, which may suggest the activation of STAT3 signalling pathway in this injury (See the arrows in A–C). One day after the induction of transient intraocular hypertension, a small amount of pSTAT3-positive cell nuclei was observed in the central region of the optic nerve. Significant increase of p-STAT3-immunolabeled signal was detected throughout the optic nerve at 2 days and was markedly decreased at 3 and 7 days (D–H). Counting of p-STAT3 -positive nuclei was shown in I. Western blot revealed that both STAT3 and pSTAT3 were mildly expressed in the optic nerve of control rats. PSTAT3 expression was substantially increased at 2 days after transient intraocular hypertension and this increase was markedly attenuated but still higher than control optic nerve at 7 days. Expression of STAT3 kept relative consistent before and after the induction of injury (J). See the quantitative analysis in K and L. Double immunofluorescence labeling for pSTAT3 and GFAP in optic nerve sections in control and Day 2 post-ischemic reperfusion injury. (Blue: DAPI; Green, anti-phosphorylated STAT3; Red, anti-GFAP) (M).

In control optic nerve, STAT3 immunoreactivity was observed in the processes and perinuclear cytoplasm of astrocyes. At post-injury day 2, nuclear translocation of STAT3 in the astrocytes was noticed, which suggested activation of STAT3 signalling pathway in response to this intraocular hypertension injury. In control optic nerve, p-STAT3 signal was essentially undetectable. At post-injury day 1, a small cluster of p-STAT3-labeled nuclei was observed in the central region of the optic nerve. Significant increase of p-STAT3-positive nuclei was noticed throughout the section of optic nerve at day 2, and was remarkably attenuated at day 3. At post-injury day 7, p-STAT3 immunohistochemistry in the optic nerve was indistinguishable from that of the controls. Double immunofluorescence labeling for pSTAT3 and GFAP showed that the expression of pSTAT3 was located in nuclei of GFAP positive cells, which suggested the activation of STAT3 signaling pathway in astrocytes in the optic nerve. The upregulation pattern of pSTAT3 was further confirmed by western blot analysis. Comparing with low level of p-STAT3 expression in control optic nerve, there is a pronounced increase of p-STAT3 level at post-injury day 2 and was then subsided gradually through day 7. Expression of total STAT3 in the optic nerve remained relatively consistent through the tested time window. (Fig. 4)

### 5. Increased expression of nestin in STAT3-positive astrocytes in the optic nerve after induction of transient intraocular hypertension

**Figure 5:**
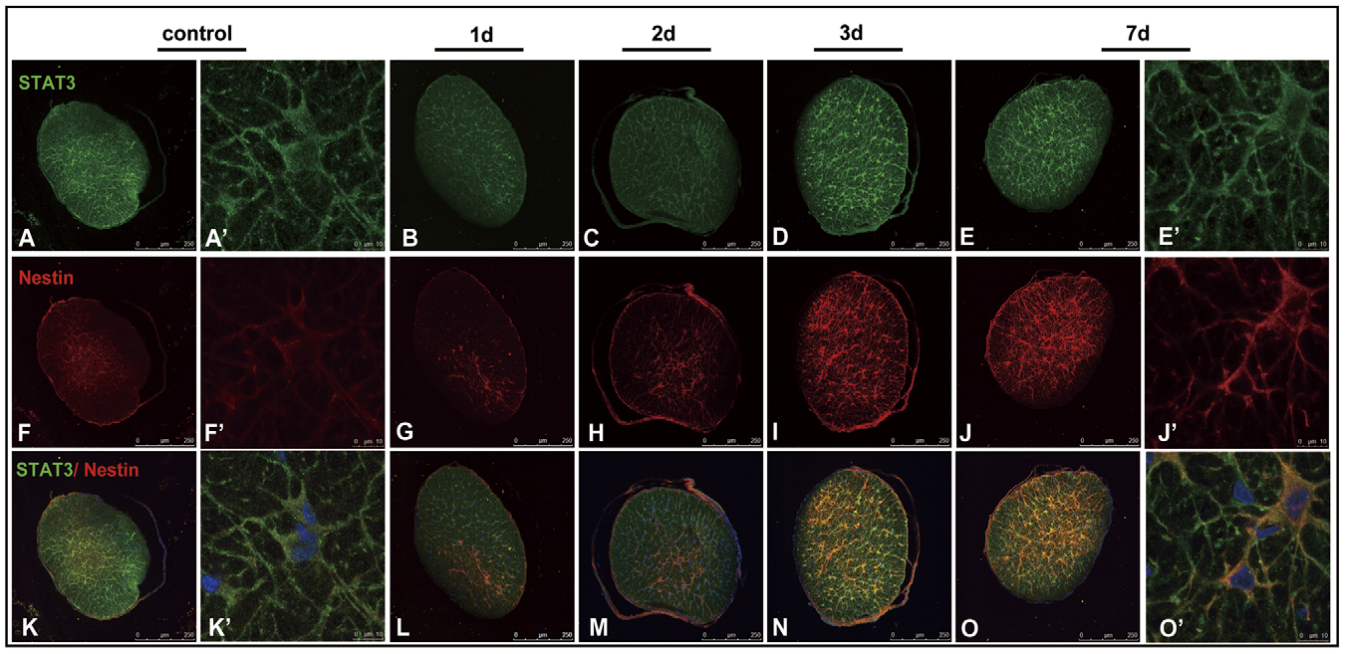
Co-expression of nestin and STAT3 in the reactive astrocytes in the optic nerve after transient intraocular hypertension injury. In control rats, only a few weakly stained nestin-positive cells were observed in the central part of the optic nerve (A,F,K). Nestin-positive signal was seen in the perinuclear cytoplasm and processes of the astrocytes, which showed good co-localization with STAT3 in the same cells (A′,F′,K′). Transient intraocular hypertension induced significant increase of nestin at 3 and 7 days (B–E,G–J,L–O). Strong nestin-positive astrocytes were seen throughout the trans-section of optic nerve (I,J,N,O). Nestin-expressing astrocytes were found co-expressing STAT3 (L,M,N,O,O′).

In control rats, some weakly stained nestin-positive cells identified as undifferentiated astrocytes were detected in the central region of optic nerve. Nestin immunoreactivity was mainly localized in the perinuclear cytoplasm and processes of these astrocytes. Nestin-postive cell bodies and processes showed good overlapping with STAT3 signals in the same cells. A substantial increase in nestin immunoreactivity was noted throughout the whole optic nerve at post-injury day 3 and 7. Nestin-positive astrocytes connected with each other forming a glial network. Essentially all cells that expressed detectable levels of nestin were co-expressed STAT3. (Fig. 5)

## Discussion

This study demonstrates a different response of astrocytes in the optic nerve from that of retina in a rat model of retinal ischemia-reperfusion. STAT3 is expressed in the soma and processes of GFAP-positive astrocytes in optic nerve. Activation of STAT3 signalling pathway by phosphorylation occurred coincident with or preceding the early response of astrocytes. STAT3 may be a key factor that mediates the activation of astrocytes in the optic nerve head in early phase of injury.

Astrocytes respond to all forms of CNS insults such as infection, trauma, ischemia and neurodegenerative disease by a process commonly referred to as reactive astrogliosis [23]–[26]. Reactive astrogliosis is a complex and multifaceted process that can involve a finely gradated continuum of changes ranging from subtle and reversible alterations in gene expression and morphology up to the pronounced and long-lasting changes associated with scar formation [27]. Recent studies provide compelling evidence that reactive astrogliosis may exert both beneficial and detrimental effects on neurons. Astrogliosis can protect CNS cells and tissue in various ways, such as uptaking of potentially excitotoxic glutamate [28], [29], [30], producing glutathione to reduce oxidative stress [30]–[32], releasing adenosine [33]. On the other hand, there is also evidence from both clinical and experimental studies that, under specific circumstances, reactive astrocytes have the potential to exert detrimental effects [34]–[36]. Astrocytes in the optic nerve head assume an activated status in glaucoma that is likely to have a profound impact on the pathological process of the axons [37]. However, the behaviour and function of the normal and activated astrocytes in optic nerve are still not well understood. In this study, we found astrocytes in retina and in optic nerve responded differently to ischemia-reperfusion injury. In retina, the expression of GFAP increased remarkably after injury which is consistent with other reports [38], [39]. However, in the present study we found in the control optic nerve, GFAP expressed at a concern level consistently, and no significant increase was observed after the injury. The quantitative analysis of the immunostaining and western blot showed GFAP levels remained stable. In the further study of astrocyte morphological changes in optic nerve, we found that IOP elevation causes a progressive change in astrocyte morphology in regions that correspond to sites of axonal injury. The most significant change was seen post-injury day 7. This finding is consistent with previous work which has demonstrated that astrocytes have the ability to undergo rapid morphological changes by redistribution of cytoskeletal proteins without any quantitative alteration to GFAP levels [40]. This difference in glial response to transient IOP elevation between retina and optic nerve may indicate different characteristics and functions of the astrocytes located in these two regions. Meanwhile, although only 1 hour IOP elevation was manipulated, we observed an early onset but sustain and progressive glial reactivity in this rat model. These evidences suggest that, once been triggered, reactive astrocytes may continuously exert impacts to surrounding neurons even the IOP has returned to its normal range. Due to the intimate histological relationship between astrocytes and RGC axons within the optic nerve, architecture changes of the glial network may directly put mechanical compression to the axons that passing through, and therefore, obstruct the axonoplasma transport of necessary molecule to the RGC. These findings may partly explain why some patients with glaucoma experience progressive cupping and visual field loss even though the IOPs have been well controlled. Better understanding of the behavior and underlying signaling mechanisms of the reactive astrocytes in optic nerve may lead to novel glia-targeting strategies and bring new insight into the glaucoma treatment in clinic.

STAT3 is a member of the Janus kinase-signal transducers and activators of transcription (Jak-STATs) signaling family that transduces signals for many cytokines and growth factors, including interleukin-6 (IL-6), IL-10, epidermal growth factor (EGF), transforming growth factor-a (TGF-a), leukemia inhibitory factor (LIF), and CNTF [41]–[45]. STAT3 is expressed by astrocytes, neurons, and other cell types in CNS [46], [47]. Recently, it has been reported that STAT3 play a Critical role in the regulation of astrogliosis and scar formation in Spinal Cord Injury [48]. Intrathecally injection of AG490, a STAT3 inhibitor, significantly reduced the nerve injury-induced STAT3 translocation in GFAP+ astrocytes in the dorsal horn and finally showed obvious recovery from allodynia [20]. These evidences suggest that STAT3 signaling pathway may play an important role in astrocyte activation in the CNS. In normal retina, STAT3 signal could be detected in the cytoplasm of both the RGCs and müller cells. After chronic IOP elevation or retina ischemia-reperfusion injury, increased STAT3 level and activated STAT3 expression (phospho-STAT3, pSTAT3) in the nucleus of RGCs and muller cells are observed, suggesting a participation of STAT3 in the neuraonal and glial responses to injury [49], [50], [51]. A previous work has shown that STAT3 is activated in several cell types including retinal neurons during photoreceptor degeneration [52]. Till now, STAT3 expression in optic nerve has not been investigated. For the first time we described the expression pattern of STAT3 in the normal and injured optic nerve. STAT3 signals are exclusively expressed in the processes and soma of GFAP-positive astrocytes under normal condition. The immunocytochemical detection of GFAP has been a standard marker for reactive astrocytes. However, GFAP delineates only less than 15% of the total volume of an astrocyte [53], [54]. It may not reflect the gross morphology of an astrocyte and may have difficulty identifying many morphological types of astrocytes [55]. STAT3 can not only reveal the finer processes but also delineate the astrocytes bodies. Regarding the co-expression of STAT3 and GFAP, STAT3 may be a more reliable marker to illustrate the astrocytes morphology and structure.

In present study, the activation of STAT3 in response to ischemia reperfusion injury in optic nerve is prior to the initial react of astrocytes. In the ischemic reperfusion model used for these studies, activation of astrocytes and axon damage were taken in from post-injury day 2 and remained elevated until day 7. In contrast, nuclear translocation of STAT3 (an indication of activation of STAT3 signaling pathway) in some population of the astrocytes in optic nerve was observed at 1 day and 2 days after the transient intraocular hypertension. Antibody of pSTAT3, the activated form of STAT3, gave a consistent result of this change. Dot and nuclear-like pSTAT3 signal was first seen in the optic nerve cross-section at 1 day post-injury and peaked at 2 days, then gradually decreased to normal range. Since activation of STAT3 occurs much earlier than the observable morphological changes of astrocyte, it may suggest an early step in astrocyte activation. Based on these data, we propose that STAT3 may mediate the activation of astrocyte in optic nerve injury. Given its early involvement in the astrocytes activation cascade, STAT3 may be a potential target for pharmacologic modulation of gliosis.

The STAT3 protein can be activated by a variety of signals, including cytokines, growth factors and oxidative stress [56]. The JAK/STAT families are known as downstream molecules of LIF and CNTF [57]. In both ischemic optic neuropathy and IOP elevation animal models, alterations of LIF, IL-6 and other JAK-STAT signaling pathway components in the optic nerve have been reported [58], [59]. These factors may lead to nuclear translocation of STAT3, and therefore, induce transcriptional changes in astrocytes, which subsequently trigger the proliferation process of these cells. In this study we revealed an increased nestin expression in the STAT3-positive cells 3 days post-injury. Nestin is a major developmental cytoskeletal protein that is expressed in undifferentiated astrocytes. It is substantially downregulated and replaced by GFAP in mature astrocytes [60]. Nestin could be re-induced in adult cells during pathological situations and usually is considered as a marker of de-differentiation cells, especially neuronal precursor cells [61], [62]. In this study, we found that Nestin was extensively upregulated in astrocytes starting from post-injury day 2 and kept rising throughout the tested time window. It suggests that the astrocytes may be de-differentiated to an earlier developmental phenotype. Furthermore, the reexpression of nestin may be important for the structure remodeling of reactive astrocytes.

In summary, this study reveals a different response pattern of astrocytes in the optic nerve from that of retina after retinal ischemia-reperfusion. STAT3 signaling pathway may play an important role in astrocyte activation in optic nerve after injury. STAT3 may be used as a selectable marker of astrocytes in optic nerve in addition to GFAP. Although the results from current study are suggestive, additional investigation is necessary to verify the role of STAT3 in astrocyte activation. For example, functional studies using inhibitor of STAT3, or constitutively activated forms of STAT3, could help determine the function of STAT3 in optic nerve astrocytes activation and retinal ganglion cell survival. The crucial role that STAT3 may play in astrocytes activation may enlighten new targets for retinal ganglion cell protection.
